# Methanolysis of Poly(lactic Acid) Using Catalyst Mixtures and the Kinetics of Methyl Lactate Production

**DOI:** 10.3390/polym14091763

**Published:** 2022-04-26

**Authors:** Fabio M. Lamberti, Luis A. Román-Ramírez, Andrew P. Dove, Joseph Wood

**Affiliations:** 1 School of Chemical Engineering, University of Birmingham, Edgbaston, Birmingham B15 2TT, UK; fxl876@student.bham.ac.uk; 2 Division of Chemical and Energy Engineering, London South Bank University, 103 Borough Road, London SE1 0AA, UK; romanral@lsbu.ac.uk; 3 School of Chemistry, University of Birmingham, Edgbaston, Birmingham B15 2TT, UK; a.dove@bham.ac.uk

**Keywords:** methanolysis, poly(lactic acid), chemical recycling, zinc acetate dihydrate, magnesium acetate tetrahydrate, 4-(dimethylamino)pyridine, triazabicyclodecene, alcoholysis, dual catalysts

## Abstract

Polylactic acid (PLA) is a leading bioplastic of which the market share is predicted to increase in the future; its growing production capacity means its end-of-life treatment is becoming increasingly important. One beneficial disposal route for PLA is its chemical recycling via alcoholysis. The alcoholysis of PLA leads to the generation of value-added products alkyl lactates; this route also has potential for a circular economy. In this work, PLA was chemically recycled via methanolysis to generate methyl lactate (MeLa). Four commercially available catalysts were investigated: zinc acetate dihydrate (Zn(OAc)_2_), magnesium acetate tetrahydrate (Mg(OAc)_2_), 4-(dimethylamino)pyridine (DMAP), and triazabicyclodecene (TBD). Dual catalyst experiments displayed an increase in reactivity when Zn(OAc)_2_ was paired with TBD or DMAP, or when Mg(OAc)_2_ was paired with TBD. Zn(OAc)_2_ coupled with TBD displayed the greatest reactivity. Out of the single catalyst reactions, Zn(OAc)_2_ exhibited the highest activity: a higher mol% was found to increase reaction rate but plateaued at 4 mol%, and a higher equivalent of methanol was found to increase the reaction rate, but plateaued at 17 equivalents. PLA methanolysis was modelled as a two-step reversible reaction; the activation energies were estimated at: *Ea*_1_ = 25.23 kJ∙mol^−1^, *Ea*_2_ = 34.16 kJ∙mol^−1^ and *Ea*_-2_ = 47.93 kJ∙mol^−1^.

## 1. Introduction

Polylactic acid (PLA) makes up a growing 18.9% of the bioplastic market. PLA is defined as a bioplastic, as it is biodegradable and its feedstock is a renewable resource [1]. Although PLA has comparable tensile strength and tensile modulus to fossil-based plastics, it is limited by its low ultimate strain, its high gas permeation, and its relatively expensive production cost [2,3,4]. Despite these limitations, PLA is still a promising polymer with the potential to replace poly(styrene) (6% of the total plastic production) as a more environmentally friendly material [5]. By using blowing agents such as CO_2_, it is possible to manufacture low-density expanded PLA as a sustainable alternative to expanded poly(styrene) [6].

In practice, the rate of biodegradation of PLA in the environment is relatively slow; after one year in the ocean, PLA only biodegrades with a mass loss of approximately 8% [7]. However, in a controlled composting environment with high temperature and humidity, PLA fully degrades in less than 90 days [8]. The main disadvantage of biodegrading PLA is that the imbedded energy of the polymer’s molecular structure is lost. Disposal routes that retain the polymer’s molecular structure include mechanical and chemical recycling methods. Comparing the life cycle assessment of these disposal routes, mechanical recycling shows the lowest environmental impact, followed by chemical recycling and composting [9]. On the other hand, mechanically recycling PLA causes the degradation of its mechanical properties, reducing the polymer’s chain length and limiting the number of times that PLA can be mechanically recycled [10]. Low-grade PLA that can no longer be mechanically recycled could be chemically recycled instead of composted. Chemical recycling allows for the generation of value-added products, or to recover monomers that can subsequently be used for the synthesis of virgin polymer [11,12].

Chemical recycling is a term used to describe a variety of chemical processes, which convert plastic waste into monomers or directly into other value-added products. Depending on the polymer type, each chemical recycling method will have advantages and disadvantages. The more common chemical depolymerisation methods for PLA include pyrolysis, hydrolysis, and alcoholysis. Pyrolysis is generally not favored, as it has a relatively high activation energy (*E_a_*) = 119 kJ∙mol^−1^ [13]. Recycling PLA via hydrolysis generates the monomer lactic acid (LA) as the product; this route avoids the relatively expensive purification costs required to produce LA from glucose fermentation [14,15]. Furthermore, LA production from the hydrolysis of PLA has a lower carbon footprint; the energy required to generate LA from the fermentation of corn glucose has been estimated as 55 MJ∙kg^−1^ of LA produced, versus only 14 MJ∙kg^−1^ of LA produced via hydrolysis [16]. 

Arguably, a more attractive chemical recycling route is alcoholysis, which generates the value-added product alkyl lactate (AL). ALs are versatile green solvents that are biodegradable and have low toxicity. ALs have the potential to replace many fossil-based chemicals in applications, such as pharmaceuticals, agriculture, food, coating, cosmetic industries, plasticizers, and solvents [3,17,18]. Depending on the alcohol nucleophile used, different ALs are formed, methanol (MeOH) produces methyl lactate (MeLa); ethanol produces ethyl lactate, propanol produces propyl lactate, etc. Alcoholysis adds value to the PLA supply chain; the market price for ethyl lactate is almost double that of PLA [19,20]. It is also possible to convert ALs to lactide, which allows for a circular PLA production after chemical recycling via alcoholysis [21,22]. Life cycle assessments have shown the alcoholysis of PLA to have clear environmental benefits when compared to hydrolysis or incineration [23].

Several catalysed processes for the alcoholysis of PLA have been reported. For example, DuPont depolymerised PLA into various ALs using H_2_SO_4_ as the catalyst, while Whitelaw et al. reported the mild methanolysis of PLA using Zr(IV)/Hf(IV)-Salalen complexes [24,25]. The alcoholysis of PLA at 50–130 °C using Zn Schiff-based complexes has also been reported in MeOH, ethanol, propanol, and butanol [26,27]. Thus, showing the versatility of alcoholysis to produce various ALs. Several studies have used ionic liquids as catalysts for the alcoholysis of PLA. These catalysts have been reported to have a high activity towards alcoholysis as well other desirable features, such as strong solvent power for organic and inorganic compounds, non-volatility, good thermal stability, and a high level of reusability [28,29,30]. It is also well reported in the literature that the metal acetates zinc acetate dihydrate (Zn(OAc)_2_), and magnesium acetate tetrahydrate (Mg(OAc)_2_), as well as the organocatalysts 4-(dimethylamino)pyridine (DMAP), and triazabicyclodecene (TBD), which are all effective catalysts for transesterification [31,32,33,34,35,36,37]. Furthermore, the recent literature investigated it using dual catalysts (Lewis acid-base pairs) for polyester recycling and found that they outperformed single catalysis [38,39,40]. A synergistic effect has been reported for Zn(OAc)_2_ coupled with DMAP, resulting in an increased polyester depolymerization rate [39,40]. These Lewis acid-base pairs were prepared by simple physical interactions, allowing for dual catalyst systems to be a scalable process relevant to the industry [38].

The aim of this work was to further investigate the effect of different commercially available catalysts and reaction conditions including catalyst loading, MeOH concentration, stirring speed, and temperature on the overall rate of methanolysis. Reaction kinetic modeling was carried out by fitting a series reaction model with a reversible second step to the concentration profiles, Arrhenius plots were derived from the variable temperature experiments. Four commercial catalysts were investigated: Zn(OAc)_2_, Mg(OAc)_2_, TBD, and DMAP. These catalysts were studied individually and in mixtures; the increased reactivity displayed with Lewis acid-base pairs could be significant for the scaling up of the process for industrial application.

## 2. Materials and Methods

### 2.1. Materials and Apparatus

PLA pellets supplied by NatureWorks (Ingeo™ 6202D, per specification weight average molecular weight 44350 g∙mol^−1^) were used without pre-treatment. Previous work concluded that the rate of degradation of PLA is independent of molecular weight, thus only one molecular weight was used for the experiments [26]. All reactants were HPLC grade; methanol (MeOH) ≥ 99.8%, and tetrahydrofuran (THF) ≥ 99.8% were purchased from Fisher Scientific, Loughborough UK. Zn(OAc)_2_, Mg(OAc)_2_, TBD, and DMAP were purchased from Sigma-Aldrich, Gillingham UK. All chemicals were used as received. Helium CP grade (≥99.999% purity), nitrogen (oxygen-free, ≥99.998%) and argon (≥99.998%) were purchased from BOC, Woking, UK.

PLA methanolysis was carried out in a 300 mL stirred autoclave with oil filled heating jacket (Parr model 4566, SciMed, Stockport, UK). The reactor temperature was controlled by a refrigerated and heating circulator (IKA CBC5-Control, Oxford, UK), connecting an oil bath to the reactor’s jacket. 

### 2.2. Procedure for Experiments Reported in Section 3.1

The procedure for the process optimization of PLA methanolysis using Zn(OAc)_2_ experiments was as follows: 2 g of PLA, 2 mol% of Zn(OAc)_2_ (relative to mol of PLA), and THF were added to the autoclave, which was then sealed and degassed with N_2_ for 5 min. The amount of THF depended on the amount of MeOH; enough THF was added so that each reaction volume was 50 mL total. Afterward, the temperature was brought to 130 °C for a further 10 min to ensure that all the PLA pellets had dissolved. Several stirring speeds were tested (0 rpm, 300 rpm, 600 rpm). Various amounts of MeOH (5.6 mL ≈ 5 equivalents, 10 mL ≈ 9 equivalents, 15 mL ≈ 13 equivalents or 19 mL ≈ 17 equivalents) in different runs were then fed into the reactor via an HPLC pump at a rate of 10 mL∙min^−1^. Reaction samples were taken periodically and tested via Agilent 6890N gas chromatograph (GC, Agilent Cheadle, UK).

### 2.3. Procedure for Experiments Reported in Section 3.2

The procedure for PLA methanolysis using mixed catalysts was as follows: 2 g PLA, various ratios of catalysts (Zn(OAc)_2_, Mg(OAc)_2_, TBD and DMAP) always totaling 2 mol% (relative to mol of PLA), and either 40 mL or 31 mL of THF (depending on MeOH amount) to make up the reaction volume to 50 mL, was added to the autoclave, which was then sealed and degassed with N_2_ for 5 min. Afterwards, the temperature was brought to 130 °C for a further 10 min to ensure that all the PLA pellets had dissolved. Two stirring speeds were tested: 300 rpm or 600 rpm. Two MeOH amounts were tested; 10 mL ≈ 9 equivalents and 19 mL ≈ 17 equivalents, which were fed into the reactor via an HPLC pump at a rate of 10 mL∙min^−1^. Reaction samples were taken periodically and tested via gas chromatograph (GC).

### 2.4. Procedure for Experiments Reported in Section 3.3

The procedure for PLA methanolysis using Zn(OAc)_2_ described was as follows: 2 g of PLA, 2 mol% of Zn(OAc)_2_, and 31 mL of THF were added to the autoclave, which was then sealed and degassed with N_2_ for 5 min. A stirring speed of 600 rpm was used. A range of temperatures were investigated 90–130 °C. Once the reactor had reached the desired temperature, 19 mL ≈ 17 equivalents of MeOH were fed into the reactor via an HPLC pump at a rate of 10 mL∙min^−1^. Reaction samples were taken periodically and tested by ^1^H NMR spectroscopic analysis.

### 2.5. GC and NMR Spectroscopy

Methyl lactate (MeLa) concentration was assessed by a GC coupled with a Flame-Ionization Detector (FID) (Agilent Technologies, 6890N, Cheadle, UK). Samples were injected by an autosampler (Agilent Technologies, 7683B. Cheadle, UK), to a 30 m × 0.32 mm ID, 0.25 µm film thickness HP-5 Agilent capillary column using helium as a carrier and make-up gas with the following conditions: inlet temperature of 150 °C, 1 µL injection volume, 1:400 split ratio, 250 °C detector temperature, with an initial oven temperature of 65 °C (held for 4 min), then 100 °C∙min^−1^ ramp to 195 °C (held for 1 min), followed by 100 °C∙min^−1^ ramp to 230 °C (held for 5 min). The initial flow rate was 0.8 mL∙min^−1^ (held for 5 min), then 100 mL∙min^−1^ ramp to 3 mL∙min^−1^ (held for 5 min). A multiple-point external standard calibration curve was prepared using standard solutions covering the range of MeLa concentrations. A linear response of the detector was determined for MeLa (R^2^ = 0.998). 

^1^H NMR spectra were measured using a 400 MHz Bruker Avance II spectrometer(Bruker Coventry UK). Samples were dissolved in CDCl_3_ and chemical shifts were referenced against tetramethylsilane (TMS). The experiments were monitored by determining the relative concentrations of methine functional groups calculated from NMR spectra. The methine protons were in one of three different environments: internal methine (Int) (*δ* = 5.09–5.21 ppm), chain-end methine (CE) (*δ* = 4.30−4.39 ppm/5.09−5.21 ppm), or MeLa methine (*δ* = 4.23−4.29 ppm). Selectivity and yield of MeLa as functions of temperature are presented, as well as the estimated kinetic parameters of the reaction.

### 2.6. Kinetic Modelling

The ^1^H NMR spectroscopic data were modeled using the reaction mechanism shown in Equation (1), previously discussed in Reference [26]. The alcohol nucleophile was in excess so was not included in the model. In Equation (1), the internal methine protons along the PLA chains are represented by (*Int*), the chain-end methine protons of the oligomer fragments are represented by (*CE*), and the methyl lactate methine protons of the product are represented by (*MeLa*). The differential Equations (2)–(4) were solved in MATLAB. PLA was depolymerized through a two-step reaction, with the second step being reversible. The coefficient *k*_1_ represents the random attack of an ester linkage by a MeOH nucleophile; each cleavage results in the generation of two *CE* oligomers. The coefficient *k*_2_ represents the forward equilibrium step, which is the formation of the product *MeLa* from *CE* oligomers; this step occurs when MeOH attacks an ester linkage of an oligomer adjacent to its *CE*. The reverse equilibrium step represented by coefficient *k-*_2_, occurs when the alcohol group of *MeLa* attacks an ester linkage of the *CE* oligomer, and itself becomes a larger oligomer.

(1) $$Int\;\overset{k_{1}}{\to }\;CE\;\overset{k_{2}}{\underset{k_{-2}}{⇄}}\;\;MeLa$$

(2) $$\frac{d\left[Int\right]}{dt}=-k_{1}\left[Int\right]$$

(3) $$\frac{d\left[CE\right]}{dt}=k_{1}\left[Int\right]-k_{2}\left[CE\right]+k_{-2}\left[MeLa\right]\;$$

(4) $$\frac{d\left[MeLa\right]}{dt}=k_{2}\left[CE\right]-k_{-2}\left[MeLa\right]$$

## 3. Results and Discussion

### 3.1. PLA Methanolysis Using Zn(OAc)_2_

**Figure 1:**
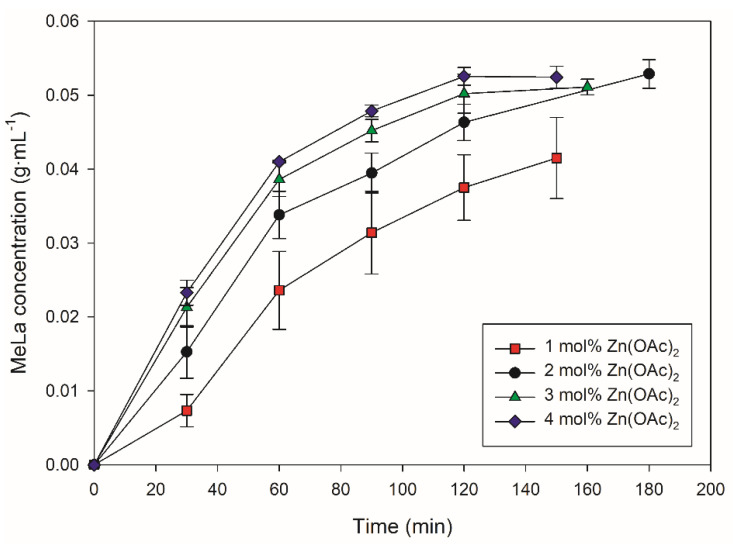
Methanolysis of 2 g of PLA at 130 °C, 300 rpm and 9 equivalents of MeOH. Effect of mol% of Zn(OAc)_2_ (Relative to mol of PLA) on the MeLa concentration (g∙mL^−1^) vs. Time (min).

These experiments were carried out to optimize PLA methanolysis in the Parr reactor; parameters such as catalyst loading, stirring speed and MeOH molar equivalents were explored. It was decided to use Zn(OAc)_2_ for these optimization experiments, as the literature often reports Zn(OAc)_2_ as having the best performance among metal acetates [41]. First, the effect of catalyst loading on the MeLa concentration was investigated as shown in Figure 1. Methanolysis was carried out at 300 rpm. A higher mol% of Zn(OAc)_2_ resulted in shorter reaction times in order to reach a MeLa concentration of >0.05 g∙mL^−1^. Increasing the catalyst loading from 1 mol% to 2 mol% resulted in the largest increase of MeLa production rate. Increasing the catalyst loading from 2 mol% to 3 mol% also increased the MeLa production rate but less so, while changing the loading from 3 mol% to 4 mol% increased the MeLa production rate the least. A higher mol% of Zn(OAc)_2_ resulted in a smaller standard error between the repeats (2–4 repeats) for each experiment, probably due to human error, as weighing out smaller amounts of catalyst has more inaccuracy. For the mixed catalyst experiments, it was therefore decided to use 2 mol% of the catalyst, as it was assumed that the other catalysts would behave similarly in terms of catalyst loading and their effect on reactivity. Moreover, 2 mol% loading of Zn(OAc)_2_ was a balance between using the least amount of catalyst, while still obtaining the higher MeLa production rate from higher loadings. 

In order to further optimize the methanolysis of PLA using Zn(OAc)_2_, the effect of stirring speed on the rate of MeLa production was studied (The range of stirring speeds tested are shown in Figure S1). A higher stirring speed of 600 rpm resulted in a MeLa concentration of >0.05 g∙ml^−1^ in the shortest times, likely owing to better dispersion of catalyst throughout the vessel, improved rates of mixing, and mass transfer. A higher stirring speed also resulted in a smaller standard error between the repeats (2–4 repeats) for each experiment. Even without stirring (at 0 rpm), the reaction reached completion at 4 h. It was assumed that the other catalysts would behave similarly in terms of stirring speed and its effect on reactivity, so it was decided to use 600 rpm for the mixed catalyst experiments. 

**Figure 2:**
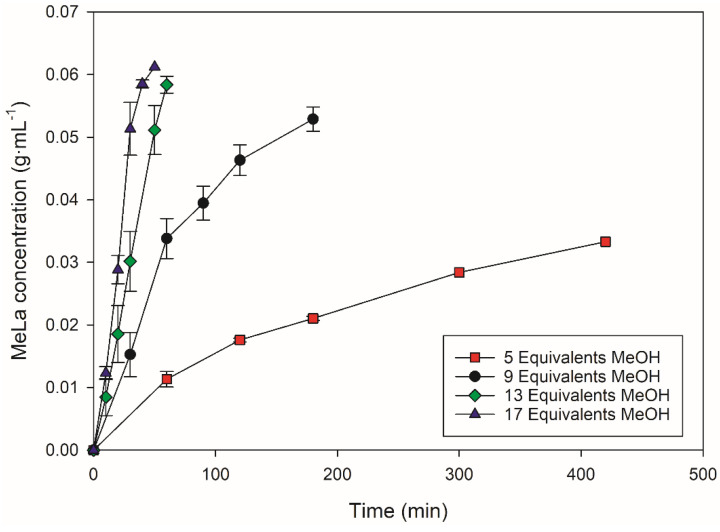
Methanolysis of 2 g of PLA at 130 °C, 300 rpm and 2 mol% Zn(OAc)_2_. Effect of MeOH molar equivalents (Relative to mol of ester bonds) on the MeLa concentration (g∙mL^−1^) vs. Time (min).

The final parameter investigated to optimize the reaction was the molar equivalent of MeOH, Figure 2. A higher equivalent of MeOH resulted in shorter reaction times in order to reach a MeLa concentration of >0.05 g∙mL^−1^. Increasing the molar equivalents of MeOH from 5 to 9 resulted in the largest increase in MeLa production rate. Increasing the equivalents from 9 to 13 also increased the MeLa production rate but by a smaller amount, while increasing the equivalents from 13 to 15 increased the MeLa production rate the least. The classic Lewis acid mechanism for transesterification using Zn(OAc)_2_, involves the polarization of an ester carbonyl group to the Zn^2+^ center, which helps facilitate the nucleophilic attack [35]. Another study reported that Zn(OAc)_2_ initiates transesterification through a mechanism that involves the initial coordination of the alcohol nucleophile to the metal center, followed by a carboxylate shift and coordination to the ester group [42]. This mechanism could explain the results that a higher equivalent of MeOH results in greater reactivity. Since Zn(OAc)_2_ coordinates the alcohol nucleophile, it could be reasoned that a higher equivalent of MeOH means Zn(OAc)_2_ will have more MeOH molecules in closer proximity, thus increasing the probability of coordination and overall reactivity. This reasoning could also be used to explain why increasing the equivalents of MeOH up to 17 causes the increase in MeLa concentration to plateau. At 17 equivalents, Zn(OAc)_2_ is fully saturated with MeOH molecules in close proximity; increasing the number of MeOH molecules beyond this limit does not increase the probability of coordination. 

### 3.2. PLA Methanolysis Using Mixed Catalysts 

It was decided to test four commercial catalysts for the methanolysis of PLA using the optimized parameters. Mg(OAc)_2_ was selected as it would allow for a good comparison with Zn(OAc)_2_. DMAP and TBD were also chosen as both organocatalysts have been reported to be effective for transesterification. Table 1 shows the results for the Methanolysis of PLA using the selected catalysts. Each catalyst was tested at both 9 and 17 equivalents of MeOH and stirring speeds of 300 and 600 rpm. Comparing the catalysts at 9 equivalents of MeOH: Zn(OAc)_2_ and TBD displayed the highest average initial rate of MeLa production (both 5.37 × 10^−4^ g∙mL^−1^∙min^−1^), followed by Mg(OAc)_2_ (5.39 × 10^−5^ g∙mL^−1^∙min^−1^), and then DMAP (3.09 × 10^−5^ g∙mL^−1^∙min^−1^). Comparing the catalysts at 17 equivalents of MeOH and 300 rpm: Zn(OAc)_2_ again displayed the highest average initial rate of MeLa production (1.42 × 10^−3^ g∙mL^−1^∙min^−1^), followed by TBD (5.27 × 10^−4^ g∙mL^−1^∙min^−1^), Mg(OAc)_2_ (9.09 × 10^−5^ g∙mL^−1^∙min^−1^), and DMAP (4.65 × 10^−5^ g∙mL^−1^∙min^−1^). TBD is the only catalyst that did not display an increase in rate of MeLa production when the equivalent of MeOH was increased. Of the four catalysts Zn(OAc)_2_ exhibited the largest increase in rate of MeLa production when the equivalent of MeOH was increased. When increasing the stirring speed from 300 to 600 rpm at 17 equivalents of MeOH both Zn(OAc)_2_ and DMAP displayed a decrease in rate of MeLa production, whereas Mg(OAc)_2_ and TBD showed an increase in rate of MeLa production. However, at these conditions Zn(OAc)_2_ again displayed the highest rate of MeLa production (1.19 × 10^−3^ g∙mL^−1^∙min^−1^), closely followed by Mg(OAc)_2_ (1.09 × 10^−3^ g∙mL^−1^∙min^−1^), then TBD (6.43 × 10^−4^ g∙mL^−1^∙min^−1^), and DMAP significantly slower (2.03 × 10^−5^ g∙mL^−1^∙min^−1^).

It was decided to investigate the effect of catalyst mixtures on the rate of methanolysis. The motivation for this was to find catalyst pairs that have enhanced reactivity in comparison to either catalyst alone. This would unlock the potential to exploit the enhanced reactivity from dual-catalyst systems for industrial chemical recycling, as a greater rate of MeLa production makes PLA alcoholysis more economically feasible. Table 2 shows the results for Methanolysis of PLA using multiple catalysts. In each reaction only 2 mol% total of catalyst was used, all catalysts were dissolved homogenously in the solvent. The initial rate of MeLa production for the dual catalyst Zn(OAc)_2_/TBD experiment (1.34 × 10^−3^ g∙mL^−1^∙min^−1^) was greater than the rate of MeLa production for the Zn(OAc)_2_ experiment (1.19 × 10^−3^ g∙mL^−1^∙min^−1^) in Table 1 at the same conditions. The higher rate could be explained by a Lewis acid-base interaction between the two catalysts, this interaction increases the activation of PLA ester carbonyls which helps facilitate the nucleophilic attack needed for depolymerisation [39]. A faster rate is also seen for the dual catalyst Zn(OAc)_2_/DMAP experiment which had a higher MeLa production rate (1.29 × 10^−3^ g∙mL^−1^∙min^−1^) than Zn(OAc)_2_ alone (1.19 × 10^−3^ g∙mL^−1^∙min^−1^), the faster rate for the dual experiment could again be explained by a Lewis acid-base interaction that aids the reaction.

The Lewis acid-base interaction is not present for the dual Zn(OAc)_2_/Mg(OAc)_2_ experiment which had a slower MeLa production rate (6.87 × 10^−4^ g∙mL^−1^∙min^−1^) than Zn(OAc)_2_ alone (1.19 × 10^−3^ g∙mL^−1^∙min^−1^), or Mg(OAc)_2_ alone (1.09 × 10^−3^ g∙mL^−1^∙min^−1^). Likewise, the beneficial Lewis acid-base interaction is not present for the dual TBD/DMAP experiment; its MeLa production rate (2.84 × 10^−4^ g∙mL^−1^∙min^−1^) was slower than the MeLa production rate for TBD alone (6.43 × 10^−4^ g∙mL^−1^∙min^−1^). If the p*K*_a_ difference between the two catalysts is great enough then proton transfer occurs, forming a stable acid-base complexion capable of enhancing the reaction [38]. As Zn(OAc)_2_ and Mg(OAc)_2_ have a similar p*K_a_* (4.54 and 8 respectively) no stable complexion forms, which explains why the dual Zn(OAc)_2_/Mg(OAc)_2_ experiment displayed a slower MeLa production rate than Zn(OAc)_2_ alone. Likewise, TBD and DMAP have a similar p*K*_a_ (15.2 and 9.6 respectively) so no stable acid-base complexion forms, thus the dual TBD/DMAP experiment had a slower MeLa production rate than TBD alone.

The dual catalyst Mg(OAc)_2_/TBD experiment displayed a higher rate of MeLa production (1.36 × 10^−3^ g∙mL^−1^∙min^−1^) compared to Mg(OAc)_2_ alone (1.09 × 10^−3^ g∙mL^−1^∙min^−1^) in Table 1 at the same conditions. Mg(OAc)_2_ and TBD have a great enough difference in p*K_a_* (8 and 15.2 respectively) to form a stable acid-base complexion. This complexion enhances the reactivity, which is why the dual Mg(OAc)_2_/TBD experiment showed a higher MeLa production rate than Mg(OAc)_2_ alone. However, the enhancing catalyst complexion is not present for the dual catalyst Mg(OAc)_2_/DMAP experiment, which displayed a slower MeLa production rate (8.44 × 10^−4^ g∙mL^−1^∙min^−1^) than Mg(OAc)_2_ alone (1.09 × 10^−3^ g∙mL^−1^∙min^−1^). As Mg(OAc)_2_ and DMAP have a similar p*K_a_* (8 and 9.6 respectively) no stable catalyst complexion can form. None of the experiments that use three or four catalysts displayed higher rates when compared to dual catalyst experiments.

### 3.3. Conversion, Selectivity, and Yield of MeLa 

**Figure 3:**
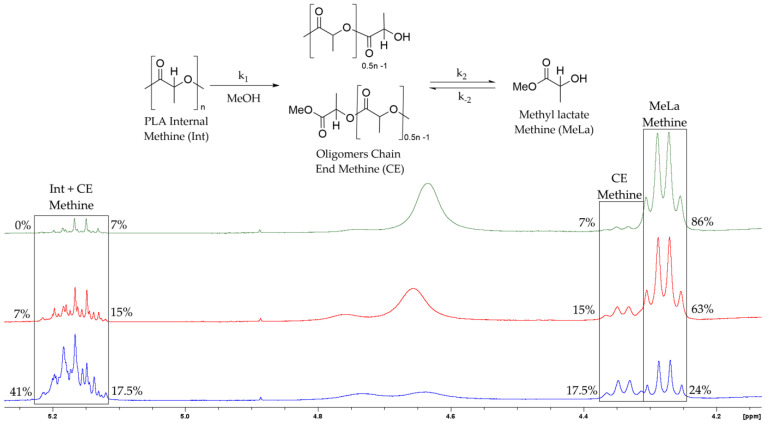
^1^H NMR (CDCl_3_, 400 MHz) stacked spectra of a methanolysis reaction at 120 °C and the relative percentage of each methine proton Int, CE and MeLa. (Blue spectrum 10 min, Red spectrum 40 min, Green spectrum 90 min).

Out of the four catalysts, Zn(OAc)_2_ produced the highest concentration of MeLa in the shortest time when tested individually, further studies were performed using Zn(OAc)_2_ alone to investigate the reaction kinetics. According to Equation (1), there are three possible environments for methine functional groups during the reaction: Int (5.09–5.21 ppm), CE (4.30–4.39 ppm/5.09–5.21 ppm), or MeLa (4.23–4.29 ppm). This enabled the determination of the reaction progress, by monitoring the relative concentration of each methine environment via ^1^H NMR spectroscopy. Reaction samples were dissolved in CDCl_3_. Figure 3 shows the stacked spectra of a methanolysis experiment at 120 °C, the relative concentration of each methine environment is displayed at 10 min, 40 min, and 90 min.

Conversions of Int groups (*X_Int_*), MeLa selectivity (*S_MeLa_*), and MeLa yield (*Y_MeLa_*) were calculated according to Equations (5)–(7),

(5) $$X_{Int}=\frac{Int_{0}-Int}{Int_{0}}$$

(6) $$S_{MeLa}=\frac{MeLa}{Int_{0}-Int}$$

(7) $$Y_{MeLa}=S_{MeLa}X_{Int}$$

*Int*_0_ is the initial concentration of the Int groups (100%). Conversion selectivity and yield of *MeLa* were calculated at 60 min. Averages for *X_Int_*, *S_MeLa_* and *Y_MeLa_* at each temperature were determined to allow for an easier comparison, as shown in Table 3. When comparing the averages, there is a clear trend that a higher temperature results in a higher Int conversion, a higher *MeLa* selectivity, and a higher *MeLa* yield. The result concurs with the Arrhenius model; a higher temperature increases the average kinetic energy of the reactant molecules, hence a larger proportion of molecules will overcome the activation energy barrier to form the product MeLa. At 130 °C the average Int conversion is 100% and the average *MeLa* selectivity and yield is 81%, the remaining 19% are CE oligomers.

### 3.4. Arrhenius Temperature-Dependent Parameters

**Figure 4:**
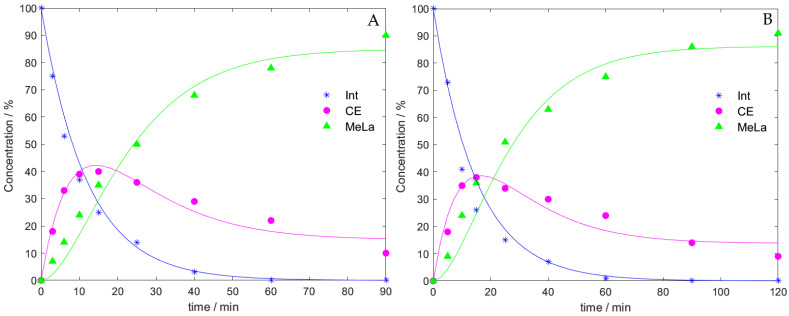
Reaction profiles obtained from ^1^H NMR spectroscopic data for methanolysis fitted in MATLAB. (**A**) 130 °C (**B**) 120 °C.

^1^H NMR spectroscopy was used to determine the relative concentrations of Int, CE, and MeLa methine groups during each experiment. The concentrations were fitted to the kinetic model described in Equation (1), the resulting rate equations were solved numerically in MATLAB producing estimates for the rate coefficients. Two typical reaction profiles are shown in Figure 4. Figure 4a highlights that methanolysis at 130 °C generates maximum concentration of 40% for CE intermediates at 15 min, while 100% conversion of Int groups is reached at 60 min. Figure 4b shows that methanolysis at 120 °C produces a maximum concentration of 39% for CE intermediates at 15 min, while 100% conversion of Int groups is not reached until 90 min. The resulting rate coefficients *k_1_* = 0.08433, 0.06672 (min^−1^), *k_2_* = 0.06757, 0.06492 (min^−1^), and *k_-2_* = 0.01184, 0.01037 (min^−1^), for 130 °C and 120 °C respectively (Table S1 in Supplementary Material shows fitted rate coefficients at all temperatures investigated). Both reaction profiles show good fits for the experimental data to the kinetic model.

**Figure 5:**
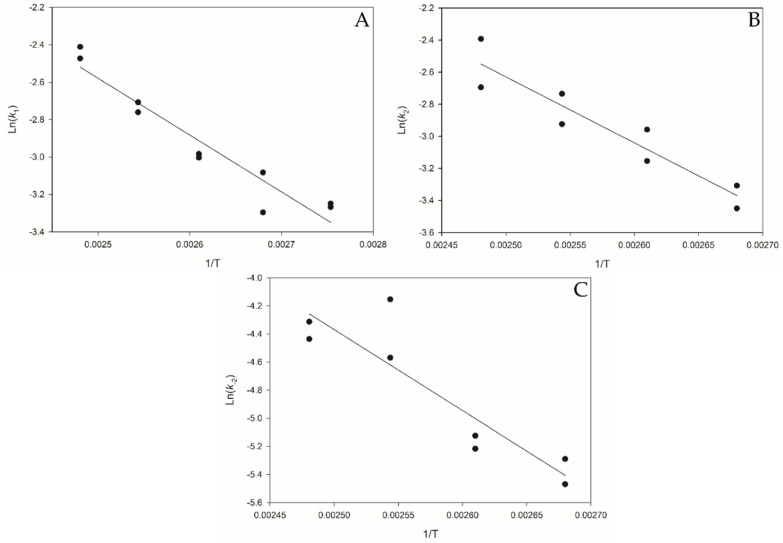
Arrhenius plots for Methanolysis of 2 g PLA, at 600 rpm, 17 equivalents MeOH and 2 mol% Zn(OAc)_2_. (**A**) = k_1_ **y** = −3035.9x + 5.01 **R^2^** = 0.9176. (**B**) = k_2_ **y** = −4111.2x + 7.6487 **R^2^** = 0.8867. (**C**) = k_−2_ **y** = −5767.6x + 10.05 **R^2^** = 0.8147.

The rate coefficients were used to generate the Arrhenius plots shown in Figure 5. The Arrhenius plots Figure 5B and C only include the temperature range 100–130 °C as this produced the best fit. The activation energies for each reaction step were estimated as *Ea*_1_ = 25.23 ± 6.16 kJ∙mol^−1^; *Ea*_2_ = 34.16 ± 12.2 kJ∙mol^−1^ and *Ea*_-2_ = 47.93 ± 22.84 kJ∙mol^−1^. The estimated activation energies highlight that *Ea*_1_ has the smallest barrier for the initial cleavage of a PLA chain to an intermediate CE. As *k_1_* > *k_2_*, PLA chains are rapidly converted to CE oligomers which then slowly forms the product MeLa, step 2 is the rate determining step of the overall reaction. Since *Ea*_-2_ has a higher barrier than *Ea*_2_, it indicates that the reverse reaction MeLa to CE occurs slower than CE to MeLa; the equilibrium lies further to the right confirmed by the maximum relative concentration of MeLa reaching approximately 90% at reaction completion.

Comparisons for the estimated *Ea*_1_ in this study can be made with literature values. Song et al. reported the methanolysis of PLA using ionic liquid 1-butyl-3-methylimidazolium acetate ([Bmim][Ac]) as the catalyst, depolymerisation was considered to proceed by first-order kinetics with an activation energy of 38.29 kJ∙mol^−1^ [28]. Also reported is the methanolysis of PLA using [Bmim][OAc]-Zn(OAc)_2_, proceeding by first-order kinetics but with a lower activation energy 20.96 kJ∙mol^−1^ [29]. The higher reactivity in the presence of the Lewis acid Zn(OAc)_2_ is likely caused by the enhanced activation of the PLA carbonyls, making the polymer more susceptible to nucleophilic attack [3,39]. Similar to the research in this paper, methanolysis has also been reported using commercially available metal-based catalysts. FeCl_3_ was found to be the most activating, achieving a 87% conversion to MeLa in 4 h at 130 °C, the first-order activation energy was reported at 32.41 kJ∙mol^−1^ [30]. Our results estimated *Ea*_1_ = 25.23 kJ∙mol^−1^ which is lower than some of the above literature values. Although ionic liquid [Bmim][OAc]-Zn(OAc)_2_ has a lower activation energy, its scalability is limited by its high costs and viscosity making it less feasible for industry uses in comparison to Zn(OAc)_2_ [3,43].

## 4. Conclusions

The methanolysis of PLA was carried out using four commercially available catalysts: Zn(OAc)_2_, Mg(OAc)_2_, TBD and DMAP. When tested individually, Zn(OAc)_2_ exhibited the highest catalytic activity. For Zn(OAc)_2_ methanolysis, a higher mol% was found to increase the reaction rate, but plateaued at 4 mol%; increasing the equivalent of MeOH was found to increase the reaction rate but plateaued at 17 equivalent. The activation energies were estimated to be: *Ea*_1_ = 25.23 ± 6.16 kJ∙mol^−1^, *Ea*_2_ = 34.16 ± 12.2 kJ∙mol^−1^ and *Ea*_-2_ = 47.93 ± 22.84 kJ∙mol^−1^. For mixed catalyst reactions, an enhancing polymer activation was found when Zn(OAc)_2_ was coupled with TBD or DMAP, or when Mg(OAc)_2_ was coupled with TBD. A great enough difference in p*K*_a_ for the dual catalysts is required to form a stable catalyst complexion; this complexion can enhance the reaction. Further research is needed to fully explore synergistic Lewis acids-base pairs; an understanding of their coordination and mechanism is required in order to fully exploit dual-catalysts systems for enhanced chemical recycling. The chemical recycling of PLA via alcoholysis is a promising end-of-life solution, adding value to the PLA supply chain through the generation of value-added ALs.

## Figures and Tables

**Table 1:** Methanolysis of 2 g PLA at 130 °C and 2 mol% of catalyst, 9–17 equivalents MeOH. Repeats (3–4 repeats) were averaged.

2 mol% Catalyst	Speed (rpm)	Molar Equivalents of MeOH	Average Final Time (min)	Average Final MeLa Concentration (g∙mL^−1^)	Average Initial Rate of MeLa Production at 40 min (g∙mL^−1^∙min^−1^)
ZnAc	300	9	173	0.0538	5.37 × 10^−4^
ZnAc	300	17	48	0.0593	1.42 × 10^−3^
ZnAc	600	17	70	0.0577	1.19 × 10^−3^
DMAP	300	9	360	0.0437	3.09 × 10^−5^
DMAP	300	17	340	0.0510	4.65 × 10^−5^
DMAP	600	17	200	0.0257	2.03 × 10^−5^
MgAc	300	9	360	0.0449	5.39 × 10^−5^
MgAc	300	17	107	0.0562	9.09 × 10^−5^
MgAc	600	17	83	0.0624	1.09 × 10^−3^
TBD	300	9	160	0.0501	5.37 × 10^−4^
TBD	300	17	140	0.0534	5.27 × 10^−4^
TBD	600	17	135	0.0557	6.43 × 10^−4^

**Table 2:** Methanolysis of 2 g PLA at 130 °C, 600 rpm, 2 mol% catalyst total and 17 eq MeOH. Repeat (2–4 repeats) were averaged.

Catalyst (2 mol% Total)	Average Final Time (min)	Average Final MeLa Concentration (g∙mL^−1^)	Average Initial Rate of MeLa Production at 40 min (g∙mL^−1^∙min^−1^)
Zn(OAc)_2_/TBD (1:1)	60	0.0584	1.34 × 10^−3^
Zn(OAc)_2_/DMAP (1:1)	80	0.0608	1.29 × 10^−3^
Mg(OAc)_2_/TBD (1:1)	80	0.0617	1.36 × 10^−3^
Mg(OAc)_2_/DMAP (1:1)	110	0.0602	8.44 × 10^−4^
TBD/DMAP (1:1)	180	0.0531	2.84 × 10^−4^
Zn(OAc)_2_/Mg(OAc)_2_ (1:1)	120	0.0561	6.87 × 10^−4^
Zn(OAc)_2_/TBD/ DMAP (1:0.5:0.5)	90	0.0600	1.27 × 10^−3^
Mg(OAc)_2_/TBD/ DMAP (1:0.5:0.5)	105	0.0591	8.72 × 10^−4^
TBD/Zn(OAc)_2_/Mg(OAc)_2_ (1:0.5:0.5)	120	0.0529	5.46 × 10^−4^
DMAP/Zn(OAc)_2_/Mg(OAc)_2_ (1:0.5:0.5)	90	0.0581	9.22 × 10^−4^
Zn(OAc)_2_/Mg(OAc)_2_/TBD/DMAP (1:1:1:1)	120	0.0626	7.41 × 10^−4^

**Table 3:** PLA methanolysis at 600 rpm with 2 mol% ZnAc. Conversion of Int groups, MeLa selectivity and MeLa yield was calculated at 60 min for different reaction temperatures.

Temperature (°C)	*X_Int_* (%)	*S_MeLa_* (%)	*Y_MeLa_* (%)	Average *X_Int_* (%)	Average *S_MeLa_* (%)	Average *Y_MeLa_* (%)
130	100	84	84	100	81	81
130	100	78	78			
120	100	68	68	99.5	72	71.5
120	99	76	75			
110	97	75	73	96	73	70
110	95	71	67			
100	92	68	63	90.5	64.5	58.5
100	89	61	54			
90	88	64	56	88	64.5	56.5
90	88	65	57			

*X_Int_*_,_*S_MeLa_***_,_** *Y_MeLa_* are determined at 60 min of reaction.

## Data Availability

Data associated with this paper are available free of charge via edata.bham.ac.uk.

## Supplementary Materials

The following supporting information can be downloaded at: https://www.mdpi.com/article/10.3390/polym14091763/s1, Figure S1. Effect of Stirring speed on the MeLa concentration and Table S1. Rate coefficients for each experiment are available to download from the publisher.

## References

[1] European Bioplastics Bioplastics Market Development Update 2021. https://docs.european-bioplastics.org/publications/market_data/2021/Report_Bioplastics_Market_Data_2021_short_version.pdf.

[2] Lee H.D., Lee M.Y., Hwang Y.S., Cho Y.H., Kim H.W., Park H.B. (2017). Separation and Purification of Lactic Acid from Fermentation Broth Using Membrane-Integrated Separation Processes. Ind. Eng. Chem. Res..

[3] Payne J., Jones M.D. (2021). The Chemical Recycling of Polyesters for a Circular Plastics Economy: Challenges and Emerging Opportunities. ChemSusChem.

[4] Farah S., Anderson D.G., Langer R. (2016). Physical and mechanical properties of PLA, and their functions in widespread applications—A comprehensive review. Adv. Drug Deliv. Rev..

[5] Geyer R., Jambeck J.R., Law K.L. (2017). Production, use, and fate of all plastics ever made. Sci. Adv..

[6] Parker K., Garancher J.P., Shah S., Fernyhough A. (2011). Expanded polylactic acid-An eco-friendly alternative to polystyrene foam. J. Cell. Plast..

[7] Haider T.P., Vçlker C., Kramm J., Landfester K., Wurm F.R. (2019). Plastics of the Future? The Impact of Biodegradable Polymers on the Environment and on Society. Angew. Chemie Int. Ed..

[8] Nampoothiri K.M., Nair N.R., John R.P. (2010). An overview of the recent developments in polylactide (PLA) research. Bioresour. Technol..

[9] Cosate de Andrade M.F., Souza P.M.S., Cavalett O., Morales A.R. (2016). Life Cycle Assessment of Poly(Lactic Acid) (PLA): Comparison Between Chemical Recycling, Mechanical Recycling and Composting. J. Polym. Environ..

[10] Schyns Z.O.G., Shaver M.P. (2021). Mechanical Recycling of Packaging Plastics: A Review. Macromol. Rapid Commun..

[11] Meys R., Frick F., Westhues S., Sternberg A., Klankermayer J., Bardow A. (2020). Towards a circular economy for plastic packaging wastes—The environmental potential of chemical recycling. Resour. Conserv. Recycl..

[12] Ragaert K., Delva L., Van Geem K. (2017). Mechanical and chemical recycling of solid plastic waste. Waste Manag..

[13] McNeill I.C., Leiper H.A. (1985). Degradation studies of some polyesters and polycarbonates-2. Polylactide: Degradation under isothermal conditions, thermal degradation mechanism and photolysis of the polymer. Polym. Degrad. Stab..

[14] VanWouwe P., Dusselier M., Vanleeuw E., Sels B. (2016). Lactide Synthesis and Chirality Control for Polylactic acid Production. ChemSusChem.

[15] Inkinen S., Hakkarainen M., Albertsson A.C., Södergård A. (2011). From lactic acid to poly(lactic acid) (PLA): Characterization and analysis of PLA and Its precursors. Biomacromolecules.

[16] Piemonte V., Sabatini S., Gironi F. (2013). Chemical Recycling of PLA: A Great Opportunity Towards the Sustainable Development?. J. Polym. Environ..

[17] Pereira C.S.M., Silva V.M.T.M., Rodrigues A.E. (2011). Ethyl lactate as a solvent: Properties, applications and production processes—A review. Green Chem..

[18] Biddy M.J., Scarlata C., Kinchin C. Chemicals from Biomass: A Market Assessment of Bioproducts with Near-Term Potential. https://www.osti.gov/biblio/1244312/.

[19] Leibfarth F.A., Moreno N., Hawker A.P., Shand J.D. (2012). Transforming polylactide into value-added materials. J. Polym. Sci. Part. A Polym. Chem..

[20] Rosales-Calderon O., Arantes V. (2019). A review on commercial-scale high-value products that can be produced alongside cellulosic ethanol. Biotechnol. Biofuels.

[21] De Clercq R., Dusselier M., Poleunis C., Debecker D.P., Giebeler L., Oswald S., Makshina E., Sels B.F. (2018). Titania-Silica Catalysts for Lactide Production from Renewable Alkyl Lactates: Structure-Activity Relations. ACS Catal..

[22] De Clercq R., Dusselier M., Makshina E., Sels B.F. (2018). Catalytic Gas-Phase Production of Lactide from Renewable Alkyl Lactates. Angew. Chemie Int. Ed..

[23] Aryan V., Maga D., Majgaonkar P., Hanich R. (2021). Valorisation of polylactic acid (PLA) waste: A comparative life cycle assessment of various solvent-based chemical recycling technologies. Resour. Conserv. Recycl..

[24] Brake L.D. (1993). Preparation of Alkyl Esters By Depolymerization. U.S. Patent.

[25] Whitelaw E.L., Davidson M.G., Jones M.D. (2011). Group 4 salalen complexes for the production and degradation of polylactide. Chem. Commun..

[26] Román-Ramírez L.A., Mckeown P., Jones M.D., Wood J. (2019). Poly(lactic acid) degradation into methyl lactate catalyzed by a well-defined Zn(II) complex. ACS Catal..

[27] Lamberti F.M., Román-Ramírez L.A., Mckeown P., Jones M.D., Wood J. (2020). Kinetics of alkyl lactate formation from the alcoholysis of poly(lactic acid). Processes.

[28] Song X., Zhang X., Wang H., Liu F., Yu S., Liu S. (2013). Methanolysis of poly(lactic acid) (PLA) catalyzed by ionic liquids. Polym. Degrad. Stab..

[29] Song X., Bian Z., Hui Y., Wang H., Liu F., Yu S. (2019). Zn-Acetate-Containing ionic liquid as highly active catalyst for fast and mild methanolysis of Poly(lactic acid). Polym. Degrad. Stab..

[30] Liu H., Song X., Liu F., Liu S., Yu S. (2015). Ferric chloride as an efficient and reusable catalyst for methanolysis of poly(lactic acid) waste. J. Polym. Res..

[31] Jehanno C., Pérez-Madrigal M.M., Demarteau J., Sardon H., Dove A.P. (2019). Organocatalysis for depolymerisation. Polym. Chem..

[32] Xu S., Held I., Kempf B., Mayr H., Steglich W., Zipse H. (2005). The DMAP-catalyzed acetylation of alcohols—A mechanistic study (DMAP = 4-(dimethylamino)pyridine). Chem. A Eur. J..

[33] Otera J. (1993). Transesterification. Chem. Rev..

[34] Thomas C., Bibal B. (2014). Hydrogen-bonding organocatalysts for ring-opening polymerization. Green Chem..

[35] Reinoso D.M., Damiani D.E., Tonetto G.M. (2012). Zinc carboxylic salts used as catalyst in the biodiesel synthesis by esterification and transesterification: Study of the stability in the reaction medium. Appl. Catal. A Gen..

[36] Capelot M., Montarnal D., Tournilhac F., Leibler L. (2012). Metal-catalyzed transesterification for healing and assembling of thermosets. J. Am. Chem. Soc..

[37] Wang Z., Yang X., Liu S., Zhang H., Wang G. (2016). Magnesium acetate used as an effective catalyst for synthesizing aliphatic polycarbonates via melt transesterification process. Chem. Res. Chinese Univ..

[38] Basterretxea A., Jehanno C., Mecerreyes D., Sardon H. (2019). Dual Organocatalysts Based on Ionic Mixtures of Acids and Bases: A Step Toward High Temperature Polymerizations. ACS Macro Lett..

[39] Delle Chiaie K.R., McMahon F.R., Williams E.J., Price M.J., Dove A.P. (2020). Dual-catalytic depolymerization of polyethylene terephthalate (PET). Polym. Chem..

[40] Lamberti F.M., Ingram A., Wood J. (2021). Synergistic Dual Catalytic System and Kinetics for the Alcoholysis of Poly (Lactic Acid). Processes.

[41] Raheem A.B., Noor Z.Z., Hassan A., Abd Hamid M.K., Samsudin S.A., Sabeen A.H. (2019). Current developments in chemical recycling of post-consumer polyethylene terephthalate wastes for new materials production: A review. J. Clean. Prod..

[42] Reinoso D.M., Ferreira M.L., Tonetto G.M. (2013). Study of the reaction mechanism of the transesterification of triglycerides catalyzed by zinc carboxylates. J. Mol. Catal. A Chem..

[43] Payne J., McKeown P., Jones M.D. (2019). A circular economy approach to plastic waste. Polym. Degrad. Stab..

