# Supplementary material for: Methanolysis of Poly(lactic Acid) Using Catalyst Mixtures and the Kinetics of Methyl Lactate Production
Source: Polymers (Basel). 2022 Apr 26;14(9):1763. doi: 10.3390/polym14091763 (PMC9105383; doi:10.3390/polym14091763)
Supplement: Supplementary file 1 [file polymers-14-01763-s001.zip › polymers-1693276-supplementary.pdf]

Supplementary Material for

Methanolysis of Poly(lactic acid) Using Catalyst Mixtures and the Kinetics of Methyl Lactate Production

Fabio M. Lamberti[1], Luis A. Román-Ramírez[3], Andrew P. Dove[2], Joseph Wood[1*]

[1] School of Chemical Engineering, University of Birmingham, Edgbaston, Birmingham B15 2TT, United Kingdom

[2] School of Chemistry, University of Birmingham, Edgbaston, Birmingham B15 2TT, United Kingdom

[3] London South Bank University, 103 Borough Road, London, SE1 0AA, United Kingdom

[*] Correspondence: J.Wood@bham.ac.uk

Contents

1 Stirring speed 3

2 Rate coefficients 3

1 Stirring speed

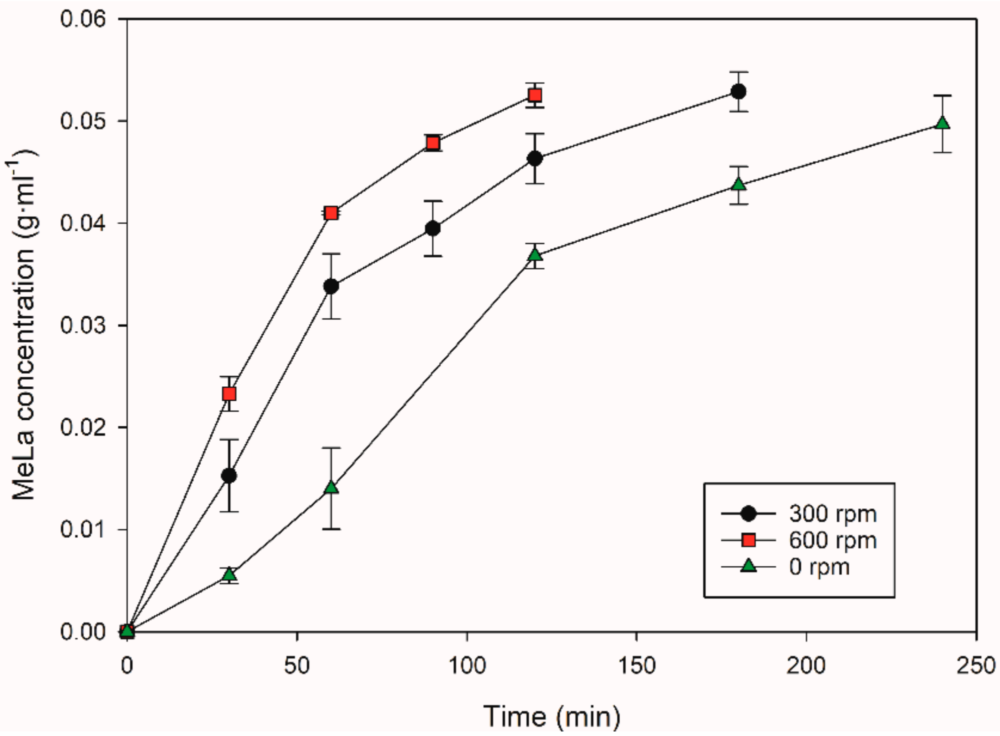

Figure S1. Methanolysis of 2 g of PLA at 130°C, 9 eq of MeOH and 2 mol% $Zn(OAc)_2$. Effect of Stirring speed on the MeLa concentration ($g{\cdot}mL^{-1}$) vs. Time (min).

2 Rate coefficients

Table S1. Rate coefficients for each experiment, catalysed by $Zn(OAc)_2$.

Temperature (°C)	k_1 (min^{-1})	k_2 (min^{-1})	k_{-2} (min^{-1})
130	0.0897	0.0914	0.0134
130	0.0843	0.0676	0.0118
120	0.0633	0.0537	0.0157
120	0.0667	0.0649	0.0104
110	0.0506	0.0519	0.0060
110	0.0496	0.0427	0.0054
100	0.0370	0.0366	0.0042
100	0.0458	0.0318	0.0050
90	0.0381	0.0425	0.0107
90	0.0388	0.0411	0.0090
